# A prediction nomogram for perineural invasion in colorectal cancer patients: a retrospective study

**DOI:** 10.1186/s12893-024-02364-9

**Published:** 2024-03-05

**Authors:** Yao Que, Ruiping Wu, Hong Li, Jinli Lu

**Affiliations:** 1 The University of South China, Hengyang, People’s Republic of China; 2 Department of General Surgery, The First People’s Hospital of Changde City, Changde, 415003 People’s Republic of China

**Keywords:** Colorectal cancer, Perineural invasion, Nomogram, Surgery, Systemic inflammatory markers

## Abstract

**Background:**

Perineural invasion (PNI), as the fifth recognized pathway for the spread and metastasis of colorectal cancer (CRC), has increasingly garnered widespread attention. The preoperative identification of whether colorectal cancer (CRC) patients exhibit PNI can assist clinical practitioners in enhancing preoperative decision-making, including determining the necessity of neoadjuvant therapy and the appropriateness of surgical resection. The primary objective of this study is to construct and validate a preoperative predictive model for assessing the risk of perineural invasion (PNI) in patients diagnosed with colorectal cancer (CRC).

**Materials and methods:**

A total of 335 patients diagnosed with colorectal cancer (CRC) at a single medical center were subject to random allocation, with 221 individuals assigned to a training dataset and 114 to a validation dataset, maintaining a ratio of 2:1. Comprehensive preoperative clinical and pathological data were meticulously gathered for analysis. Initial exploration involved conducting univariate logistic regression analysis, with subsequent inclusion of variables demonstrating a significance level of *p* < 0.05 into the multivariate logistic regression analysis, aiming to ascertain independent predictive factors, all while maintaining a p-value threshold of less than 0.05. From the culmination of these factors, a nomogram was meticulously devised. Rigorous evaluation of this nomogram's precision and reliability encompassed Receiver Operating Characteristic (ROC) curve analysis, calibration curve assessment, and Decision Curve Analysis (DCA). The robustness and accuracy were further fortified through application of the bootstrap method, which entailed 1000 independent dataset samplings to perform discrimination and calibration procedures.

**Results:**

The results of multivariate logistic regression analysis unveiled independent risk factors for perineural invasion (PNI) in patients diagnosed with colorectal cancer (CRC). These factors included tumor histological differentiation (grade) (OR = 0.15, 95% CI = 0.03–0.74, *p* = 0.02), primary tumor location (OR = 2.49, 95% CI = 1.21–5.12, *p* = 0.013), gross tumor type (OR = 0.42, 95% CI = 0.22–0.81, *p* = 0.01), N staging in CT (OR = 3.44, 95% CI = 1.74–6.80, *p* < 0.001), carcinoembryonic antigen (CEA) level (OR = 3.13, 95% CI = 1.60–6.13, *p* = 0.001), and platelet-to-lymphocyte ratio (PLR) (OR = 2.07, 95% CI = 1.08–3.96, *p* = 0.028).These findings formed the basis for constructing a predictive nomogram, which exhibited an impressive area under the receiver operating characteristic (ROC) curve (AUC) of 0.772 (95% CI, 0.712–0.833). The Hosmer–Lemeshow test confirmed the model's excellent fit (*p* = 0.47), and the calibration curve demonstrated consistent performance. Furthermore, decision curve analysis (DCA) underscored a substantial net benefit across the risk range of 13% to 85%, reaffirming the nomogram's reliability through rigorous internal validation.

**Conclusion:**

We have formulated a highly reliable nomogram that provides valuable assistance to clinical practitioners in preoperatively assessing the likelihood of perineural invasion (PNI) among colorectal cancer (CRC) patients. This tool holds significant potential in offering guidance for treatment strategy formulation.

## Introduction

Colorectal cancer (CRC) stands as a prominent driver of cancer-related occurrences and fatalities globally, holding the distinction of being the second most prevalent malignancy in adults and a substantial contributor to cancer-related mortality [1]. According to projections from the American Cancer Society, the year 2023 is expected to witness an estimated 153,020 new CRC diagnoses alongside 52,550 unfortunate fatalities attributed to the disease [2]. Treatment strategies for locally advanced colon cancer (LACC) are evolving towards preoperative neoadjuvant therapy, which has significantly improved disease-free survival (DFS) for patients by enhancing the R0 resection rate and initiating early treatment to prevent cancer metastasis [3]. Additionally, this approach offers personalized treatment options based on MMR status. According to the FOxTROT trial, neoadjuvant chemotherapy significantly reduces the risk of residual and recurrent cancer in patients with T3-T4 LACC, demonstrating its safety and efficacy. However, caution is advised when choosing neoadjuvant chemotherapy for dMMR LACC patients, as more than 50% did not exhibit a pathological response [4]. Neoadjuvant immunotherapy not only elicits significant pathological responses in dMMR LACC patients but also shows major pathological responses in a subset of pMMR LACC patients, revealing the potential of this treatment strategy to cure more patients with this type of cancer [5]. For T4 LACC patients, despite receiving adjuvant chemotherapy with fluoropyrimidine and oxaliplatin, there remains a high risk of recurrence. Therefore, it is recommended that all T4b LACC patients, regardless of dMMR or pMMR status, undergo neoadjuvant therapy to maximize treatment efficacy [6].

Tumor invasion and metastasis depend on various components within the Tumor Microenvironment (TME) [7, 8]. Recent studies have highlighted the role of neurons and axons as components of the TME in tumor invasion and metastasis, with increasing attention on the impact of nerves on other cells within the TME [9, 10]. While the roles of blood and lymphatic vessels in tumor growth and invasion are widely recognized, the role of nerves is often underestimated. Growing evidence suggests that Perineural Invasion (PNI) is a critical factor in cancer progression, significantly associated with reduced patient survival rates and increased tumor recurrence and metastasis rates [11, 12]. The term "perineural invasion" (PNI) refers to the infiltration of tumor cells into various layers of nerve walls, nerve bundle sheaths, or the encirclement of nerves by tumor cells exceeding 33% [13]. Within the colorectal cancer (CRC) patient population, the prevalence of perineural invasion (PNI) ranges between 20 and 57% [14]. PNI has emerged as a predictive factor for CRC progression or recurrence, potentially offering valuable insights for clinical practitioners when devising patient-specific treatment strategies [15, 16].

Furthermore, in accordance with the American Joint Committee on Cancer (AJCC) staging manual, perineural invasion (PNI) stands out as a specific and significant prognostic indicator for colorectal cancer [17]. The clinical practice guidelines outlined by the National Comprehensive Cancer Network (NCCN) similarly recognize PNI as a high-risk factor for postoperative recurrence in colorectal cancer and advocate for adjuvant therapy in cases of stage II colorectal cancer combined with PNI [18]. Prior investigations have established a connection between PNI status and the secretion of extracellular vesicles (EVs), as well as the expression levels of plasma miR-21 and nerve growth factor (NGF) [19–21], though their clinical utility as biomarkers remains somewhat limited.

While perineural invasion (PNI) holds significant prognostic value, its assessment is currently reliant on postoperative pathological biopsies. The preoperative identification of PNI could serve as a valuable tool for clinicians, aiding in the optimization of clinical decisions such as the necessity of neoadjuvant therapy and the adequacy of surgical resection. As such, the primary objective of this study is to construct and validate a nomogram grounded in the preoperative clinical and pathological attributes of colorectal cancer (CRC) patients. This nomogram is designed to predict the likelihood of PNI occurrence.

## Materials and methods

### Patient selection

This retrospective study protocol received approval from the Ethics Committee of the First People's Hospital of Changde, affiliated with Xiangya Medical College, Central South University. All experiments and methodologies were executed in strict accordance with applicable guidelines and regulations. The analysis encompassed a cohort of 388 patients diagnosed with colorectal cancer (CRC) who underwent surgical procedures at the Department of General Surgery of the First People's Hospital of Changde, affiliated with Xiangya Medical College, Central South University, spanning from June 2021 to June 2023.

Inclusion Criteria:1. Patients with a verified diagnosis of colorectal cancer (CRC) and tumor staging conducted in accordance with the guidelines outlined in the American Joint Committee on Cancer (AJCC-TNM Staging Manual, 8th edition)0.2. Resectable colorectal cancer lesions assessed and planned for curative resection by a multidisciplinary team (MDT)0.3. Availability of postoperative histopathological reports providing details regarding the perineural invasion (PNI) status.4. Completion of both CT plain scan and enhanced examination conducted within 7 days preceding the surgical intervention. Exclusion Criteria:1. Preoperative conditions such as severe anemia, infections, and hematologic diseases.2. Treatments to increase white blood cells or platelets conducted within one month prior to surgery.3. Patients who underwent preoperative treatments, including radiotherapy, chemotherapy, or chemoradiotherapy.4. Patients diagnosed with metastatic cancer or malignancies in other anatomical sites.5. Patients with incomplete medical records or insufficient examination data.

**Fig. 1:**
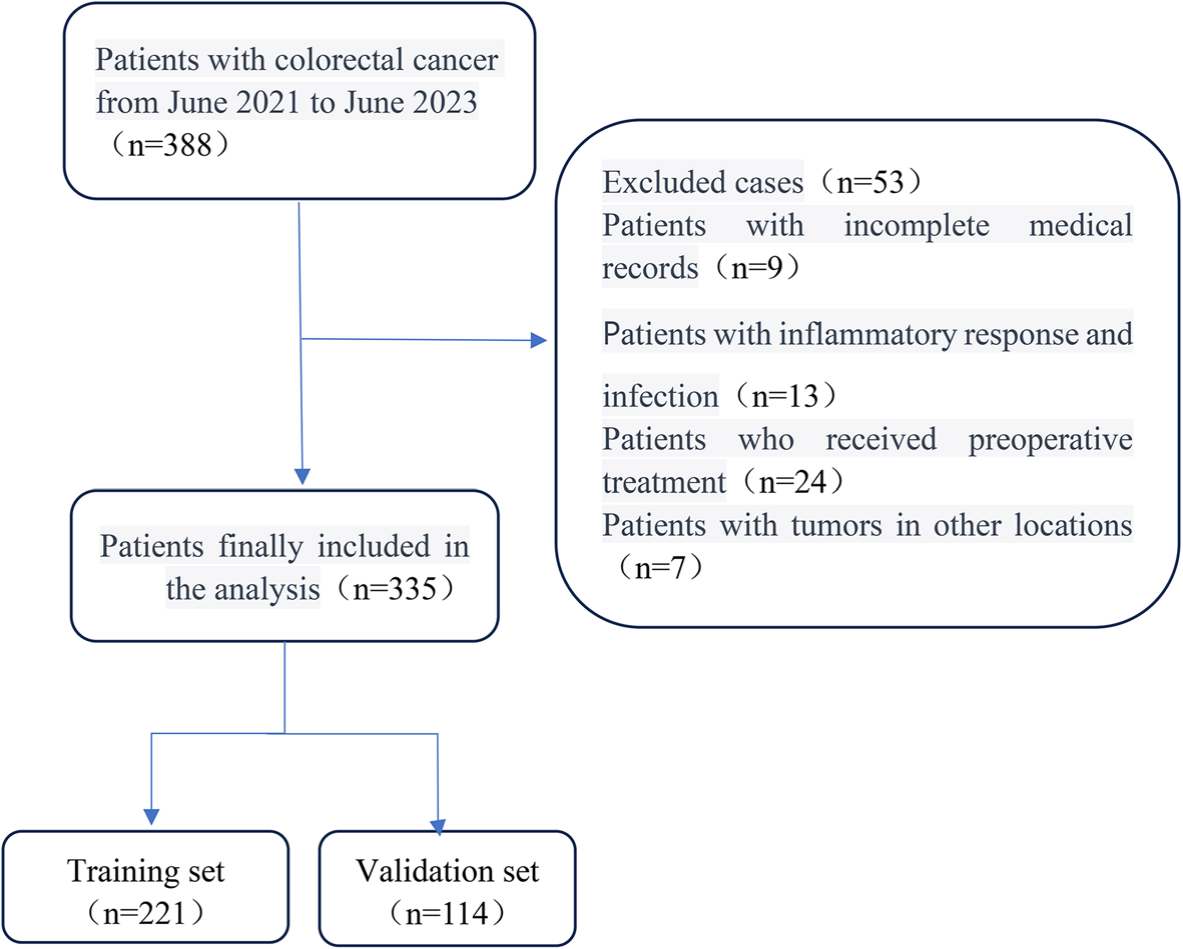
Flowchart of patient selection

Following adherence to the defined inclusion and exclusion criteria, a total of 335 patients were ultimately included in this study. These patients possessed comprehensive data and were subsequently allocated, in a 2:1 ratio, to two distinct datasets: the training group, comprising 221 cases, and the validation group, comprising 114 cases. This allocation was executed through the utilization of computer-generated random numbers, as depicted in Fig. 1.

### Data collection

The collected dataset encompassed a range of variables, including age, gender, body mass index (BMI), smoking history, serum tumor markers, complete blood count results, T-stage and N-stage determined via computerized tomography (CT) scans, preoperative histological type, histological differentiation (grading), and tumor macroscopic type. The staging of T-stage and N-stage adhered to the guidelines outlined in the 8th edition of the American Joint Committee on Cancer (AJCC) staging manual.

### Definitions

Based on the results of complete blood count tests, we calculated the ratios of neutrophil-to-lymphocyte (NLR), platelet-to-lymphocyte (PLR), and lymphocyte-to-monocyte (LMR). By constructing Receiver Operating Characteristic (ROC) curve plots, we identified the optimal cut-off values for the occurrence of Perineural Invasion (PNI). Patients were categorized into groups based on these values: NLR ≤ 2.52 and NLR > 2.52, PLR ≤ 136.98 and PLR > 136.98, LMR ≤ 2.76 and LMR > 2.76. Furthermore, based on the reference ranges of tumor markers, patients were divided into groups according to the following criteria: Carcinoembryonic Antigen (CEA) ≤ 5 ng/ml and CEA > 5 ng/ml, Carbohydrate Antigen 199 (CA199) ≤ 35 KU/L and CA199 > 35 KU/L, Carbohydrate Antigen 125 (CA125) ≤ 35 KU/L and CA125 > 35 KU/L, and Aberrant Glycosylation Protein Detection (TAP) ≤ 121 um^2^ and TAP > 121 um^2^.

### Nomogram construction and performance assessment

We conducted a thorough assessment of independent predictive factors (*p* < 0.05) within the training group through multivariate logistic regression analysis. This data was then utilized to formulate a nomogram designed for predicting the occurrence of perineural invasion (PNI) among colorectal cancer (CRC) patients. To gauge the goodness of fit between observed and predicted values in both the training and validation groups, we employed calibration curves and conducted the Hosmer–Lemeshow test.For an in-depth evaluation of the nomogram's performance in both groups, we utilized Receiver Operating Characteristic (ROC) curves and calculated the Area Under the Curve (AUC). Additionally, we carried out Decision Curve Analysis (DCA) to assess the clinical utility of the nomogram, evaluating the net benefit at various threshold probabilities within both the training and validation datasets. To enhance the robustness of our analysis, we employed the bootstrap method with 1000 iterations for sample resampling, focusing on discrimination and calibration.

### Statistical analysis

All statistical analyses were conducted utilizing R software (version 4.3.0). Logistic regression analysis, nomogram construction and calibration were executed with the rms package. Calibration curves were generated using the calibrate and val.prob functions from the rms package, while decision curve analysis (DCA) was facilitated with the rmda package.Numerical variables were reported as the median and interquartile range (IQR), and group comparisons were conducted employing the Mann–Whitney U test. Categorical variables were expressed as counts (percentages), and group comparisons were performed using $\chi 2$ tests or Fisher's exact tests. All statistical tests were two-tailed, with a significance level set at *p* < 0.05.

## Results

### Clinicopathological characteristics of the patients with colorectal cancer (CRC)

This study encompassed a total of 335 patients, and an overview of the patients' demographic characteristics is presented in Table 1. The training dataset consisted of 221 cases, with 110 males and 111 females, while the validation dataset comprised 114 cases, consisting of 54 males and 60 females. Within this cohort, there were 146 cases of rectal cancer, 105 cases of left-sided colon cancer, and 84 cases of right-sided colon cancer. As per the postoperative pathological reports, 148 patients were diagnosed with perineural invasion (PNI).

**Table 1:** Clinicopathologic characteristics of the patients with colorectal cancer (CRC)

Characteristics	Training set (*n* = 221)			Validation set (*n* = 114)		
	PNI-negative n (%)	PNI-positive n (%)	*P*-value	PNI-negative n (%)	PNI-positive n (%)	*P*-value
Gender			0.942			0.208
Female	59 (50)	52 (51)		32 (47)	28 (61)	
Male	60 (50)	50 (49)		36 (53)	18 (39)	
Age (years)			0.761			0.454
Median (IQR) (year)	60 (53, 69)	59.5 (54, 68.75)		66 (55.25, 73)	63.5 (56.25, 68.75)	
BMI (Kg/m^2^)			0.266			0.436
Median (IQR)(Kg/m^2^)	21.63 (20.24, 23.61)	21.52 (19.82, 23.05)		21.6 (20.07, 23.51)	21.05 (20.19, 22.56)	
Smoking			0.243			0.953
NO	90 (76)	69 (68)		43 (63)	28 (61)	
YES	29 (24)	33 (32)		25 (37)	18 (39)	
CEA(ng/ml)			0.006			0.371
≤ 5	59 (50)	31 (30)		41 (60)	23 (50)	
> 5	60 (50)	71 (70)		27 (40)	23 (50)	
CA199(KU/L)			0.044			0.347
≤ 35	108 (91)	82 (80)		63 (93)	40 (87)	
> 35	11 (9)	20 (20)		5 (7)	6 (13)	
CA125(KU/L)			0.127			0.391
≤ 35	113 (95)	101 (99)		66 (97)	43 (93)	
> 35	6 (5)	1 (1)		2 (3)	3 (7)	
TAP(um^2^)			0.899			0.934
≤ 121	10 (8)	10 (10)		4 (6)	2 (4)	
> 121	109 (92)	92 (90)		64 (94)	44 (96)	
NLR			0.026			0.024
≤ 2.52	47 (39)	25 (25)		30 (44)	10 (22)	
> 2.52	72 (61)	77 (75)		38 (56)	36 (78)	
PLR			0.013			0.339
≤ 136.98	53 (45)	28 (27)		31 (46)	16 (35)	
> 136.98	66 (55)	74 (73)		37 (54)	30 (65)	
LMR			0.383			0.032
≤ 2.76	39 (33)	27 (26)		24 (35)	7 (15)	
> 2.76	80 (67)	75 (74)		44 (65)	39 (85)	
CT N-stage			0.065			0.416
N0	44 (37)	25 (25)		27 (40)	14 (30)	
N1-N2	75 (63)	77 (75)		41 (60)	32 (70)	
CT T-stage			0.001			0.182
T1-T2	64 (54)	32 (31)		35 (51)	17 (37)	
T3-T4	55 (46)	70 (69)		33 (49)	29 (63)	
Primary site			< 0.001			0.008
Left colon	48 (40)	26 (25)		24 (35)	7 (15)	
Right colon	32 (27)	17 (17)		23 (34)	12 (26)	
Rectum	39 (33)	59 (58)		21 (31)	27 (59)	
Endoscopic biopsy			0.004			0.002
Poorly	10 (8)	16 (16)		5 (7)	7 (15)	
Moderately	91 (76)	83 (81)		51 (75)	39 (85)	
Well	18 (15)	3 (3)		12 (18)	0 (0)	
Pathological type			0.536			0.875
adenocarcinoma	111 (93)	98 (96)		64 (94)	44 (96)	
non-adenocarcinoma	8 (7)	4 (4)		4 (6)	2 (4)	
Tumor gross type			0.037			0.071
Ulceration	44 (37)	54 (53)		22 (32)	25 (54)	
Infiltrative	4 (3)	4 (4)		5 (7)	3 (7)	
Protruded	69 (58)	40 (39)		40 (59)	17 (37)	
Other	2 (2)	4 (4)		1 (1)	1 (2)	

*BMI* Body Mass Index, *Primary site* Primary tumor site, *Smoking* Smoking history, *CT T-stage* Tumor stage determined by CT scan, *CT N-stage* Lymph node stage determined by CT scan, *Endoscopic biopsy* Histological differentiation (grading), *Pathological type* Histological type, *Tumor gross type* Gross classification of the tumor, *CEA* Carcinoembryonic Antigen, *CA199* Carbohydrate Antigen 199, *CA125* Carbohydrate Antigen 125, *TAP* Aberrant Glycosylation Protein Detection, *NLR* Neutrophil-to-Lymphocyte Ratio, *PLR* Platelet-to-Lymphocyte Ratio, *LMR* Lymphocyte-to-Monocyte Ratio, *IQR* Interquartile Range

No statistically significant differences (*p* > 0.05) were observed between the PNI-positive and PNI-negative groups concerning age, gender, smoking history, body mass index (BMI), abnormal glycoprotein TAP, carbohydrate antigen CA125 (CA125), lymphocyte-to-monocyte ratio (LMR), and histological classification. However, noteworthy distinctions (*p* < 0.05) emerged between these two groups in terms of T-stage and N-stage in CT, tumor histological differentiation (grading), tumor macroscopic type, tumor primary location, carcinoembryonic antigen (CEA), carbohydrate antigen CA199 (CA199), neutrophil-to-lymphocyte ratio (NLR), and platelet-to-lymphocyte ratio (PLR).Multivariate logistic regression analysis identified six clinical and pathological characteristics as independent risk factors for perineural invasion (PNI) in patients with colorectal cancer (CRC). These findings are summarized as follows: tumor histological differentiation (grading) (OR = 0.15, 95% CI = 0.03–0.74, *p* = 0.02), primary tumor location (OR = 2.49, 95% CI = 1.21–5.12, *p* = 0.013), tumor macroscopic type (OR = 0.42, 95% CI = 0.22–0.81, *p* = 0.01), N staging in CT (OR = 3.44, 95% CI = 1.74–6.80, *p* < 0.001), CEA (OR = 3.13, 95% CI = 1.60–6.13, *p* = 0.001), and PLR (OR = 2.07, 95% CI = 1.08–3.96, *p* = 0.028) (as depicted in Table 2).

**Table 2:** Univariate and Multivariate logistic regression analysis of the patients with colorectal cancer (CRC)

Variable	Univariate analysis		Multivariate analysis	
	**OR (95%CI)**	***P*-value**	**OR (95%CI)**	***P*-value**
Age (years)	1.00(0.97–1.02)	0.828		
BMI (Kg/m^2^)	0.93(0.83–1.06)	0.277		
Gender Female	Reference			
Male	0.95(0.56–1.61)	0.836		
Smoking NO	Reference			
YES	1.48(0.82–2.68)	0.189		
CA125(KU/L) ≤ 35	Reference			
> 35	0.19(0.02–1.57)	0.123		
CA199(KU/L) ≤ 35	Reference			
> 35	2.39(1.09–5.28)	0.030		
CEA(ng/ml) ≤ 5	Reference			
> 5	2.25(1.29–3.92)	0.004	3.13(1.60–6.13)	0.001
TAP(um^2^) ≤ 121	Reference			
> 121	0.84(0.34–2.12)	0.718		
LMR ≤ 2.76	Reference			
> 2.76	1.35(0.76–2.43)	0.308		
PLR ≤ 136.98	Reference			
> 136.98	2.12(1.20–3.74)	0.009	2.07(1.08–3.96)	0.028
NLR ≤ 2.52	Reference			
> 2.52	2.01(1.12–3.60)	0.019		
CT N-stage N0	Reference			
N1-N2	2.55(1.46–4.42)	0.001	3.44(1.74–6.80)	< 0.001
CT T-stage T1-T2	Reference			
T3-T4	1.81(1.01–3.24)	0.047		
Endoscopic biopsy				
Poorly	Reference			
Moderately	0.57(0.24–1.33)	0.192	0.69 (0.26–1.84)	0.457
Well	0.10(0.02–0.45)	0.002	0.15 (0.03–0.74)	0.020
Pathological type				
adenocarcinoma	Reference			
non-adenocarcinoma	0.57(0.17–1.94)	0.365		
Primary site				
Left colon	Reference			
Right colon	0.98(0.46–2.09)	0.960	0.79 (0.34–1.85)	0.593
Rectum	2.79(1.49–5.22)	0.001	2.49 (1.21–5.12)	0.013
Tumor gross type				
Ulceration	Reference			
Infiltrative	0.81(0.19–3.45)	0.781	0.65 (0.14–2.91)	0.572
Protruded	0.47(0.27–0.82)	0.008	0.42 (0.22–0.81)	0.010
Other	1.63(0.28–9.33)	0.583	1.02 (0.16–6.66)	0.983

### Nomogram construction and performance

**Fig. 2:**
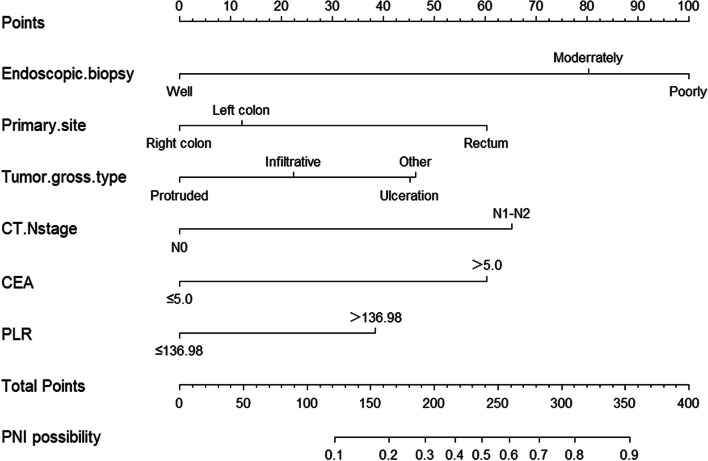
Nomogram predicting the risk of perineural invasion (PNI) in patients with colorectal cancer

Utilizing multifactorial logistic regression analysis, we identified six independent risk factors: tumor histological differentiation (grading), primary tumor location, gross tumor type, N staging in CT, carcinoembryonic antigen (CEA), and the platelet-to-lymphocyte ratio (PLR). These factors were employed to construct a nomogram (Fig. 2) for preoperatively predicting the occurrence of perineural invasion (PNI) in colorectal cancer patients.

**Fig. 3:**
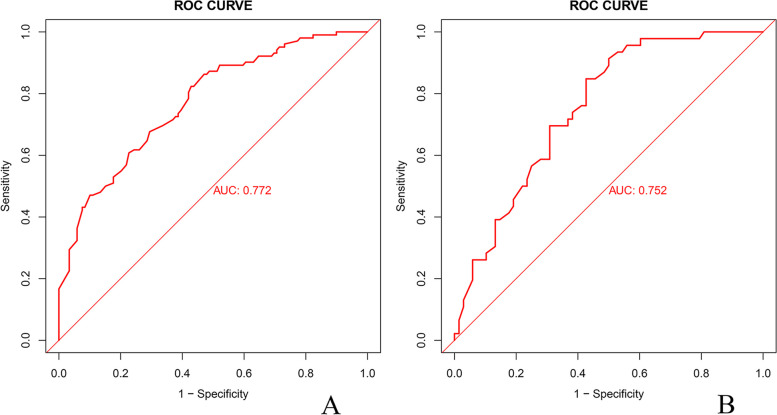
ROC curve. **A** Training group, (**B**). Validation group. *ROC* Receiver Operating Characteristic, *AUC* Area Under the ROC Curve

**Fig. 4:**
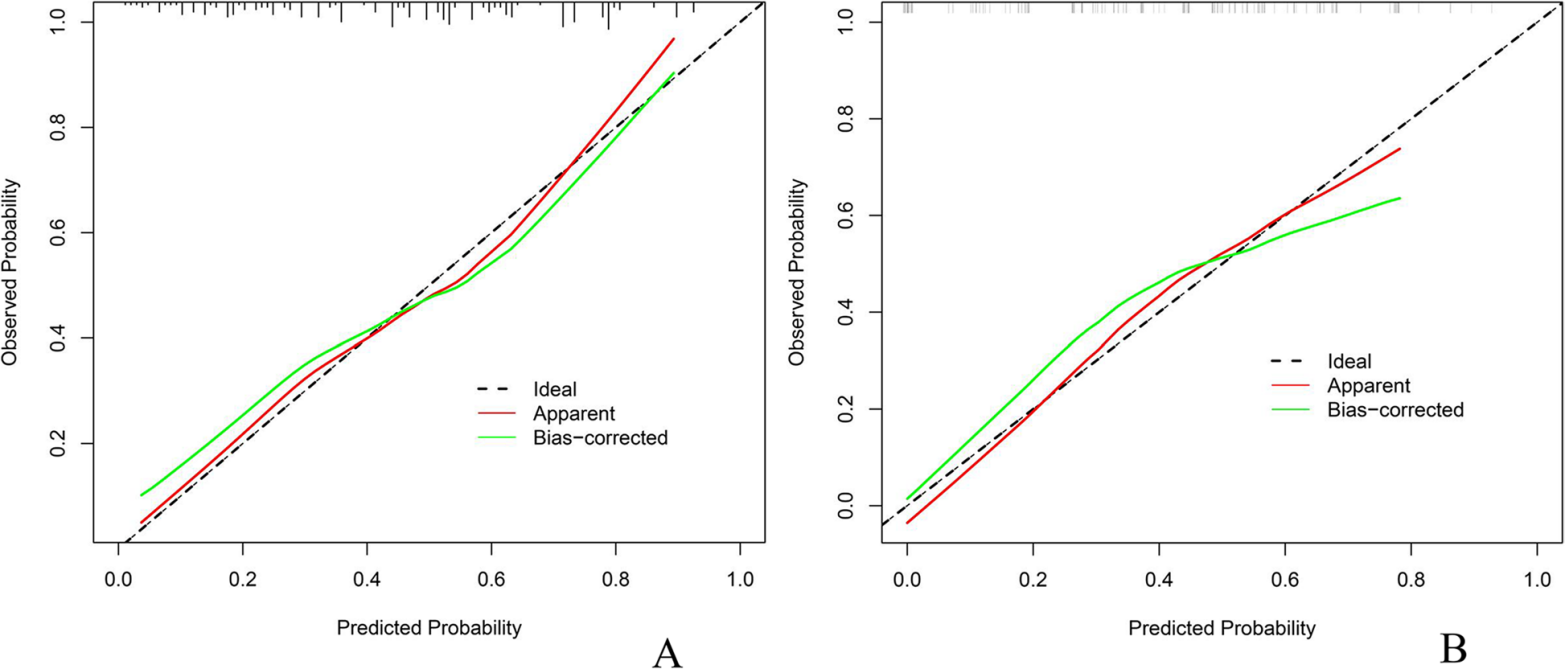
Calibration curve for predicting the possibility of PNI in colorectal cancer patients. **A** Training group, (**B**). Validation group. *PNI* Perineural Invasion

The receiver operating characteristic (ROC) curve analysis revealed an area under the curve (AUC) of 0.772 (95% CI, 0.712–0.833) for the predicted nomogram within the training group dataset and 0.752 (95% CI, 0.664–0.839) within the validation group dataset (as illustrated in Fig. 3). Moreover, the calibration curves exhibited a favorable agreement between the predicted probabilities and the actual outcomes for both the training and validation group datasets (refer to Fig. 4). The p-values obtained from the Hosmer–Lemeshow test were 0.47 and 0.72, respectively, indicating no significant deviation and confirming the model's robust fit.

**Fig. 5:**
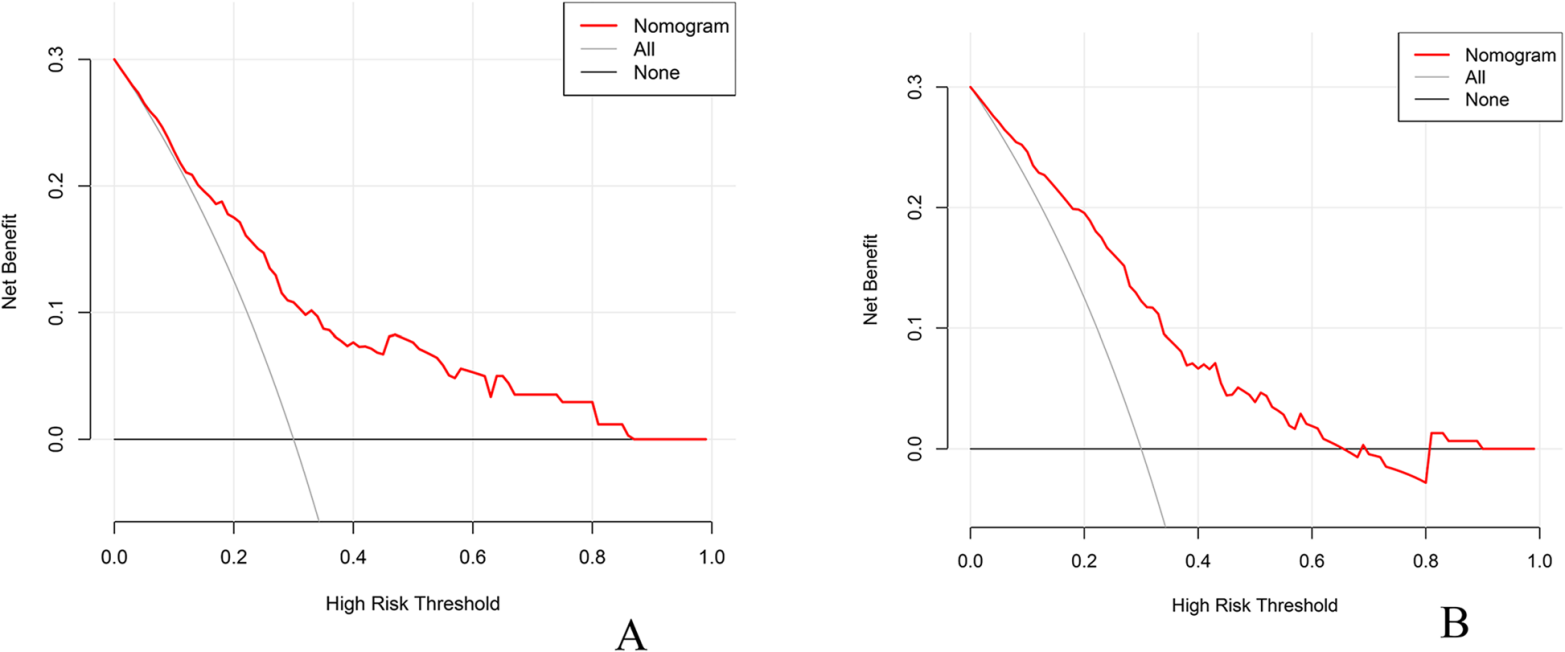
Decision curve analysis for predicting PNI in colorectal cancer patients. **A** Training group, (**B**). Validation group. *PNI* Perineural Invasion

Decision curve analysis (DCA) was conducted to assess the practical clinical utility of the nomogram in both the training and validation group datasets (refer to Fig. 5). The analysis revealed that within the training group, setting threshold probabilities between 13 and 85%, and in the validation group, between 8 and 68%, could lead to net benefits when guiding clinical interventions for patients with perineural invasion (PNI) based on the nomogram predictions. These findings underscore the nomogram's practical applicability in clinical settings for estimating the risk of perineural invasion (PNI) among colorectal cancer (CRC) patients.

### Clinical utility of the nomogram

Decision curve analysis (DCA) was systematically performed to evaluate the clinical applicability of the nomogram in both the training and validation group datasets (as displayed in Fig. 5). The analysis clearly suggests that when the threshold probabilities for the training group and validation group were strategically set within the ranges of 13% to 85% and 8% to 68%, respectively, clinical interventions guided by the nomogram's predictions had the potential to yield net benefits for patients dealing with perineural invasion (PNI).This unequivocally underscores the practical significance of the nomogram in real-world clinical contexts, particularly in forecasting the likelihood of PNI among colorectal cancer (CRC) patients.

## Discussion

Given the profound impact of perineural invasion (PNI) on cancer prognosis, the National Comprehensive Cancer Network (NCCN) guidelines and the guidelines of the Chinese Society of Clinical Oncology (CSCO) have proposed the mandatory inclusion of PNI status in the pathological reports of colorectal cancer (CRC) patients [22, 23]. The occurrence of PNI not only results in pain and functional impairment in the affected organs but also leads to postoperative local recurrence, metastasis, and infiltration due to tumor cells remaining within or around nerves, which are key factors in poor prognosis [24, 25]. The incidence of PNI varies across different stages of CRC, approximately 10% in stages I-II and up to 40% in stage IV [26]. Adjuvant chemotherapy has been shown to prolong the five-year disease-free survival of patients with stage II-III CRC and to alleviate the adverse effects of PNI on survival [27, 28]. PNI is closely related to tumor metastasis, recurrence, overall survival (OS), and disease-free survival (DFS) [29]. Lymph node invasion (LNI) is another pathological feature associated with poor disease prognosis, and some studies have suggested PNI as a predictive factor for diagnosing LNI [26, 30]. Additionally, the presence of PNI is linked to tumor lymph node metastasis [31], which may be related to frequent contact with large-diameter axons in lymph nodes. Notably, in CRC cases without nerve and lymph node invasion, there is a significant increase in the five-year survival rate of patients [32, 33]. The strong correlation between PNI and lymph node metastasis warrants further investigation.

Nomograms, which integrate a variety of significant factors for individualized risk assessment, are extensively utilized in evaluating the prognosis of colorectal cancer (CRC) patients [34–37]. By employing multifactor logistic regression analysis, we identified independent predictive factors associated with perineural invasion (PNI) in CRC patients and based on these factors, developed a nomogram. For each CRC patient, the higher the total score, the greater the risk of developing PNI. For example, consider a CRC patient with the tumor originating in the rectum (60 points), N staging in CT as N0 (0 points), gross tumor type as ulcerative (22 points), low tumor differentiation (100 points), carcinoembryonic antigen (CEA) positive (60 points), and a platelet-to-lymphocyte ratio (PLR) > 136.98 (39 points), culminating in a total of 281 points. This score correlates with an approximate 70% probability of developing PNI. Given the threshold of 50%, this patient is classified as high-risk and may be considered for neoadjuvant chemotherapy.

In our investigation, we have discerned that the platelet-to-lymphocyte ratio (PLR) (*p*= 0.028) serves as an independent predictive factor for perineural invasion (PNI). The presence of malignant tumors can incite an escalation in platelet (PLT) counts through the release of thrombopoietin (TPO) and interleukin-6 (IL-6). Consequently, heightened platelet activation can foster tumor growth and metastasis, a phenomenon recognized as the "positive feedback loop" in paraneoplastic thrombocytosis [38]. When peripheral blood lymphocyte counts diminish, the tumor burden tends to rise, thus promoting tumor dissemination and metastasis [39]. PLR, as a composite biomarker reflecting both inflammatory and immune status within the body, indicates an augmentation in the inflammatory response or a reduction in immune response. This suggests a decline in the body's anti-tumor capacity, which, in turn, results in an unfavorable prognosis and distant metastasis of tumors [40, 41]. Although an extensive body of research has illuminated the significance of increased local lymphocyte infiltration within tumors and elevated systemic inflammatory responses as pivotal clinical indicators influencing patient prognosis [42–46], studies elucidating the impact of PLR on PNI among CRC patients remain scarce. Consequently, further investigations are warranted to delve into the intricate relationship between these factors.

Serum tumor markers, which are substances secreted by tumor cells or produced by the body in response to tumor-related stimuli, play a crucial role not only in assisting the diagnosis of colorectal cancer (CRC) but also in guiding treatment, evaluating therapeutic effectiveness, and predicting outcomes. Studies have indicated that elevated preoperative levels of carcinoembryonic antigen (CEA) are an independent predictive factor for perineural invasion (PNI) in CRC patients[43]. Our research supports this finding, highlighting the significance of CEA as a marker for PNI. However, the diagnostic effectiveness of CEA alone is limited. Therefore, we recommend using CEA in conjunction with other indicators, such as the platelet-to-lymphocyte ratio (PLR), to enhance diagnostic accuracy and provide a more comprehensive evaluation of PNI risk in CRC patients. Some studies have proposed quantifying factors such as tumor histological differentiation (grading), N staging in CT (lymph node metastasis), gross tumor type, a family history of cancer in first-degree relatives, and the degree of weight loss in the three months prior to diagnosis. These factors were used to establish a preoperative nomogram for predicting perineural invasion (PNI) in colorectal cancer (CRC) patients. Additionally, another nomogram combining carcinoembryonic antigen (CEA) levels and CT radiomic features has been developed. The clinical utility of both these nomograms has been validated through decision curve analysis (DCA), confirming their applicative value in clinical practice [47, 48]. In our nomogram, poorer tumor histological differentiation (grading) and higher N staging result in higher scores, indicating a greater likelihood of perineural invasion (PNI) in colorectal cancer (CRC) patients, consistent with related studies [49, 50]. Ulcerative, infiltrative, and polypoid are common gross types of CRC. Previous research has suggested that the gross type of CRC is associated with PNI [51]. Our findings indicate that the ulcerative type is more prone to PNI compared to the polypoid type, which may be attributed to the tumor's destruction of normal tissue.

Our study found that the incidence of perineural invasion (PNI) in rectal cancer is higher than in colon cancer, consistent with related reports indicating a PNI incidence of 20.6% in rectal cancer compared to 14.1% in colon cancer [52]. Currently, no studies have demonstrated the impact of left-sided versus right-sided colon cancer on PNI. Although our research suggests that left-sided colon cancer is more prone to PNI than right-sided, it's important to note that our data come from a single center and involve a relatively small sample size, which may introduce bias. Moving forward, we aim to conduct multi-center, large-scale studies to further explore the influence of the tumor's primary location on perineural invasion (PNI).

Some research has developed a nomogram for predicting perineural invasion (PNI) in colorectal cancer (CRC) patients using CT radiomic features [53]. However, the semantic features of regions of interest (ROI) on CT images, such as tumor diameter or size measurements, are highly subjective and can vary significantly between clinicians with different levels of experience, thus introducing certain limitations. In contrast, our study, in addition to CT imaging, incorporates commonly used clinical and pathological features such as serum inflammatory markers and histopathological indicators. Our findings indicate that tissue differentiation (grading), primary tumor location, gross tumor type, N staging in CT, carcinoembryonic antigen (CEA), and the platelet-to-lymphocyte ratio (PLR) are closely related to the occurrence of PNI. These clinical and pathological features not only provide a more comprehensive assessment of CRC, but they are also readily accessible. The nomogram constructed using these six clinical-pathological features has demonstrated stability and accuracy in internal validations. Moreover, the results of decision curve analysis (DCA) suggest that our model is also valuable in guiding clinical decision-making by healthcare professionals.

While this study has its strengths, there are limitations to consider. Firstly, as a retrospective analysis including only patients who underwent curative surgery, selection bias may limit the generalizability of our findings, indicating a need for future prospective studies to validate and expand upon these results. Secondly, we assessed PNI status using HE staining, not the more precise immunohistochemistry technique based on anti-S100 antibodies [54, 55], which may affect the diagnostic accuracy for PNI-positive cases. Future research should employ more advanced techniques to enhance diagnostic precision. Thirdly, the risk factors included in our model are relatively limited and may not encompass all potential factors influencing PNI occurrence. This implies that the predictive power of the model might be restricted, and future studies should incorporate a broader range of risk factors to improve the model’s predictive capabilities. Lastly, all study participants were from a single hospital and only internal validation was conducted. To enhance the generalizability and stability of our findings, external validation using data from other hospitals is necessary.

## Conclusions

In summary, our research introduces a nomogram for preoperatively predicting the risk of perineural invasion (PNI) in colorectal cancer (CRC) patients. This tool aids clinicians in making more precise decisions and is anticipated to enhance both the staging strategies and treatment approaches for tumors. In the future, we plan to further increase the accuracy and universality of our prediction model through external validation in diverse populations and conditions. Additionally, we aim to investigate more clinical and pathological factors that may influence PNI, which will provide additional information for the personalized treatment of colorectal cancer.

## Data Availability

The work dataset supports the findings of this study are available on reasonable request from the corresponding author, and the data are not publicly available due to privacy or ethical restrictions.
